# Investigation of the Association Between Alcohol Outlet Density and Alcohol-Related Hospital Admission Rates in England: Study Protocol

**DOI:** 10.2196/resprot.6300

**Published:** 2016-12-16

**Authors:** Ravi Maheswaran, John Holmes, Mark Green, Mark Strong, Tim Pearson, Petra Meier

**Affiliations:** ^1^ Public Health GIS Unit School of Health and Related Research University of Sheffield Sheffield United Kingdom; ^2^ Sheffield Alcohol Research Group School of Health and Related Research University of Sheffield Sheffield United Kingdom

**Keywords:** alcohol, outlets, hospital, admissions, geography, epidemiology

## Abstract

**Background:**

Availability of alcohol is a major policy issue for governments, and one of the availability factors is the density of alcohol outlets within geographic areas.

**Objective:**

The aim of this study is to investigate the association between alcohol outlet density and hospital admissions for alcohol-related conditions in a national (English) small area level ecological study.

**Methods:**

This project will employ ecological correlation and cross-sectional time series study designs to examine spatial and temporal relationships between alcohol outlet density and hospital admissions. Census units to be used in the analysis will include all Lower and Middle Super-Output Areas (LSOAs and MSOAs) in England (53 million total population; 32,482 LSOAs and 6781 MSOAs). LSOAs (approximately 1500 people per LSOA) will support investigation at a fine spatial resolution. Spatio-temporal associations will be investigated using MSOAs (approximately 7500 people per MSOA). The project will use comprehensive coverage data on alcohol outlets in England (from 2003, 2007, 2010, and 2013) from a commercial source, which has estimated that the database includes 98% of all alcohol outlets in England. Alcohol outlets may be classified into two broad groups: on-trade outlets, comprising outlets from which alcohol can be purchased and consumed on the premises (eg, pubs); and off-trade outlets, in which alcohol can be purchased but not consumed on the premises (eg, off-licenses). In the 2010 dataset, there are 132,989 on-trade and 51,975 off-trade outlets. The longitudinal data series will allow us to examine associations between changes in outlet density and changes in hospital admission rates. The project will use anonymized data on alcohol-related hospital admissions in England from 2003 to 2013 and investigate associations with acute (eg, admissions for injuries) and chronic (eg, admissions for alcoholic liver disease) harms. The investigation will include the examination of conditions that are wholly and partially attributable to alcohol, using internationally standardized alcohol-attributable fractions.

**Results:**

The project is currently in progress. Results are expected in 2017.

**Conclusions:**

The results of this study will provide a national evidence base to inform policy decisions regarding the licensing of alcohol sales outlets.

## Introduction

### Purpose of Study

Availability of alcohol is a major policy issue for governments and one of the availability factors is the density of alcohol outlets in a geographic area. However, whilst numerous international research studies on alcohol outlet density have examined associations with consumption and with crime and disorder [1-4], few have examined associations with alcohol-related hospital admissions [5-9].

The costs of alcohol-related health harms that are placed upon national health services are substantial. There are an estimated 800,000 alcohol-attributable hospital admissions per year in England, and the total yearly cost of alcohol-related harm to the National Health Service (NHS) has been estimated to be approximately £2.7 billion [10,11].

Patterns in alcohol outlet density have changed in recent years in England. There has been a general decline in the number of local pubs, but this has been accompanied by an increase in off-premise outlets, including supermarket outlets. There has also been an increase in the concentration of bars in city centers. These observations are based upon unpublished work carried out in our department.

A shift is occurring in alcohol licensing policies within the United Kingdom, with increasing emphasis on controlling alcohol consumption and harm by bringing public health bodies (or considerations) into licensing decision-making [12,13]. Local authorities have been given powers to control alcohol outlet density through cumulative impact policies in England and Wales, and licensing statements in Scotland, but the consideration of chronic harms in licensing policy is currently hampered by the very limited evidence base [14].

The purpose of our study is to investigate the association between alcohol outlet density and hospital admissions for alcohol-related conditions in a national (English) small area level ecological study. We aim to determine whether correlations exist between outlet density and hospital admissions at the small area level, and to examine whether changes in outlet density over time are associated with changes in alcohol-related admissions.

The key novel aspects of our proposal include the investigation of hospital admissions for a range of alcohol-related conditions, incorporation of both cross-sectional and longitudinal analyses, the use of small geographic areas, and the examination of patterns (and changes in patterns) in the density of different types of alcohol outlets.

### Literature Review

Several studies have been carried out to investigate associations between alcohol outlet density, alcohol consumption, and harm, which have been summarized in systematic reviews [1-4]. The great majority of studies examined the effects of outlet density on alcohol consumption, several examined effects on crime and disorder, and a few examined links between outlet density and child abuse, sexually transmitted infections, and suicide [1-4]. Very few studies, however, have examined the effects of outlet density on chronic harms, which typically include conditions such as alcoholism and alcoholic liver disease.

Alcohol outlets may be classified into two broad groups: on-trade outlets, comprising outlets in which alcohol can be purchased and consumed on the premises (eg, pubs); and off-trade outlets, in which alcohol can be purchased but not consumed on the premises (eg, off-licenses). Early studies examining chronic effects of alcohol consumption used large geographic areas as the units of analysis, for example a state level analysis in the United States which found that on-trade outlet density was correlated with liver cirrhosis mortality [15]. More recently, Theall et al found an association between neighborhood-level off-trade alcohol outlet density and self-reported liver problems in Los Angeles and Louisiana [16]. Two recent studies in British Columbia found that increases in the density of off-trade outlets were associated with increases in alcohol-related mortality [17,18].

Few published studies to date have examined associations between outlet density and hospital admissions [5-9]. Alcohol-related hospital admissions are useful to study because they allow both acute and chronic effects of outlet density to be examined. Livingston, in a Melbourne study, found that on-trade outlets were strongly associated with assault-related hospital admissions (an acute effect) but were also associated with chronic alcohol-induced conditions to a lesser extent, whilst off-trade outlets were strongly associated with both assaults and chronic alcohol induced conditions [6]. Stockwell et al examined a broader group of conditions in British Columbia and found associations between off-trade outlets (private liquor stores) and hospital admissions for both acute and chronic conditions, but no significant associations were observed for on-trade outlets [7]. Tatlow et al found an association between outlet density and alcohol-related hospital admissions in San Diego County, but did not distinguish between acute and chronic conditions and did not present results separately for on-trade and off-trade outlets [5].

Regarding evidence on outlet density and harm in the United Kingdom, there are two recent studies of note [8,9]. Fone et al examined the association between alcohol outlet density and alcohol consumption, hospital admissions, accident and emergency attendances, and crime in Wales and found evidence of association with all four of these outcomes. However, this study did not differentiate between on-trade and off-trade outlets, and did not distinguish between admissions for acute and chronic conditions related to alcohol [8]. Richardson et al examined the association between on-trade and off-trade outlet density, and hospital admissions and mortality in the four largest cities in Scotland [9]. This study combined all conditions wholly attributable to alcohol, but also examined alcoholic liver disease separately as an indicator of chronic harm, and found associations with both on-trade and off-trade outlet density. This study did not examine subcategories of on-trade and off-trade outlets.

In addition, there are two online reports examining alcohol outlet density in the United Kingdom. Chiang found an association between alcohol outlet density and crime in Glasgow, but the study did not differentiate between on-trade and off-trade outlets [19]. A mapping exercise of alcohol outlet density was carried out in the East Midlands, but associations with outcomes were not examined [20]. We recently completed work on a joint Medical Research Council/Economic and Social Research Council (MRC/ESRC)-funded strategic program grant (Interdisciplinary Alcohol Policy Effectiveness Research Programme), which included an element examining alcohol outlet density and alcohol consumption.

### Theoretical Considerations and Hypotheses to Be Investigated

Robust theoretical models of the relationship between alcohol outlet density and alcohol-related harm are still being developed, and these often reflect empirical analyses by focusing on the relationship between on-trade density and acute harms or violence [21,22]. Models in which the proposed mechanism for the outlet density impact does not necessarily require high alcohol consumption (eg, where violence occurs due to collisions between drinkers exiting several densely situated on-trade outlets) are relevant for explaining acute harms [23].

Economic models suggest that alcohol outlet density may impact on chronic harms through increased consumption. A key proposition is that increased outlet density lowers the full cost of alcohol purchases by increasing average proximity to outlets, thereby reducing travel, energy, and time costs [22]. Competition between densely situated outlets may also exert downward pressure on prices, or lead to diversifications of the market, which stimulate consumption by better matching supply and demand [24]. The above theoretical considerations, in addition to previous empirical results, inform the hypotheses that we plan to investigate.

We hypothesize that on-trade outlets (specifically bars and pubs) will be associated with acute alcohol-related hospital admissions, based on the theoretical considerations outlined above, and on previous results [6]. We also expect that on-trade outlets will be associated with admissions for chronic alcohol-related conditions, based on results from previous studies [6,9].

We hypothesize that off-trade outlets will be associated with chronic alcohol-related conditions, and that this association will be most clearly observed for hospital admissions which are wholly attributable to alcohol (eg, chronic liver disease), based on the theoretical considerations outlined above and on results from previous studies [6,7,9]. We hypothesize that off-trade outlets will also be associated with acute harms, based on previous research and theories supporting this link, which includes violence related to high alcohol consumption (eg, domestic violence and local street violence) and *pre-loading*, especially due to the purchase of cheap alcohol from off-licenses and convenience stores [6].

We have previously found that alcohol-related mortality rates in England are much higher in men, reach a peak in the middle-aged adult population, and are substantially higher in the most socioeconomically deprived areas [25]. We therefore plan to investigate associations in deprived and nondeprived areas, in both men and women, and in young and older adults. We anticipate that associations will be stronger in more deprived areas and in men, and that stronger associations will be seen for acute harms in younger adults and for chronic harms in older adults. The geography of urban and rural areas is quite different, with more dispersed populations and alcohol outlets in rural areas. We therefore plan to investigate associations in urban and rural areas.

## Methods

### Study Design and Area of Study

We plan to use cross-sectional (ecological correlation) and longitudinal (cross-sectional time series) study designs. Geographic units of analysis will include all Lower and Middle Super-Output Areas (LSOAs and MSOAs) in England (53 million total population; 32,482 LSOAs; 6781 MSOAs). LSOAs (approximately 1500 people per LSOA) are the smallest spatial units at which anonymized hospital admission data are available, and will support investigation at a fine spatial resolution. Spatio-temporal associations will be investigated using MSOAs (approximately 7500 people per MSOA). Both the LSOA- and MSOA-level analyses will be at a finer spatial scale than those used in some of the previously published ecological studies on alcohol outlet density and hospital admissions. Average populations in the geographic units of analysis were approximately 17,000 and 52,000 in two studies [6,7] and >10,000 in another study (which did not provide further details) [5]. Studies in the United Kingdom used spatial scales similar to the LSOAs that we plan to use [8,9].

### Ethics Approval

The study has been approved by the University of Sheffield (School of Health and Related Research) Research Ethics Committee.

### Data on Alcohol Outlets

We purchased comprehensive coverage data on alcohol outlets in England from CGA Strategy for 2003, 2007, and 2010 for our MRC/ESRC-funded project, and are negotiating the purchase of 2013 data. CGA Strategy has estimated that the database includes 98% of all alcohol outlets in England. In the 2010 dataset, there were 132,989 on-trade and 51,975 off-trade outlets. The longitudinal data series will allow for the examination of associations between changes in outlet density and changes in hospital admission rates.

In our MRC/ESRC-funded program, we carried out a comprehensive investigation of the association between different outlet category groups and alcohol consumption, using data on the latter from the national General Lifestyle Survey [26]. We classified on-trade outlets into three categories: pubs, bars, and nightclubs; restaurants; and other premise types (which included hotels, casinos, social clubs, and sports venues). We classified off-trade outlets into two categories: supermarkets; and other off-trade outlets (which included off-licenses and convenience stores). We calculated densities of these different subcategories of outlets within 0.25 kilometers (kms), 1 km, 3 km, and 5 km of postcode centroids (>1 million residential postcodes in England). We also calculated distance to the nearest outlet within each subcategory. In addition, we calculated distances from postcode centroids to the nearest cluster of pubs, bars, and nightclubs, with a cluster defined as four or more outlets within 250 meters of each other.

The key results were (1) increasing density of pubs, bars, and nightclubs within a 1 km radius of postcode centroids was associated with increasing alcohol consumption and (2) increasing density of smaller off-trade outlets (off-licenses and convenience stores) within a 5 km radius of postcode centroids was associated with increasing alcohol consumption. We therefore plan to use these two categories of outlets and their respective distance radii as the primary exposure measures. The National Travel Survey indicates that 1 km is the average walking journey length [27], while a Competition Commission report indicates that 80-90% of consumers live within 5 km of a convenience store [28].

As the units of analysis in this proposed project are LSOAs and MSOAs, we will calculate the average outlet density (mean and variance) for postcodes within each LSOA and MSOA (there are approximately 25 postcodes per LSOA and 125 per MSOA), weighting the average by the number of domestic delivery points for each postcode as a proxy for postcode population counts [29].

We will also investigate whether the density of young people’s circuit bars and nightclubs (a subcategory within pubs, bars, and nightclubs defined by the data provider based on the demographic profile of the clientele and design of the venue, such as provision of a dance floor or low seats-to-patrons ratio) is associated with acute hospital admissions amongst teenagers and young adults, as these venues might be more likely to be associated with pub-crawl and binge drinking activities. We plan to analyze the other outlet categories as well, and anticipate that associations seen with on-trade outlets will be specific to pubs, bars, and nightclubs, and not restaurants or other types of premises. We also anticipate that associations with off-trade outlet density will be specific to off-licenses and convenience stores, and will be unlikely for supermarkets. Supermarkets are likely to serve large catchment areas, particularly if the use of home delivery is employed, and are used by most the population, making them unlikely to exert a strong local density-type effect.

### Availability of Data and Material

The data on alcohol outlets may be obtained from CGA Strategy [30]. Data on hospital admissions in England may be obtained from NHS Digital [31].

### Hospital Admissions Data

We plan to use anonymized Hospital Episode Statistics (HES) data to examine hospital admissions in England from 2003 to 2013. HES data include the primary diagnosis (International Statistical Classification of Diseases and Related Health Problems-10; ICD-10 code) for each admission. We plan to examine associations separately for acute and chronic conditions, and examine lag effects for chronic conditions. The contribution of alcohol to the etiology of numerous conditions has been investigated previously, and has resulted in internationally standardized alcohol attributable fractions (AAFs). AAFs are used by Public Health England to estimate the proportion of the burden of harm, classified by age and sex, from each condition that is attributable to alcohol [32,33]. For acute conditions, we will include conditions that are partially attributable to alcohol, as most conditions in this category have AAF<1. For chronic conditions, we will examine two categories: conditions that are wholly attributable to alcohol (ie, AAF=1), and conditions that are partially attributable to alcohol (ie, AAF<1).

The major contributors to admissions in the AAF=1 category include, “mental and behavioral disorders due to use of alcohol” (ICD-10 code F10), “alcoholic liver disease” (ICD-10 code K70), and, “chronic pancreatitis (alcohol induced)” (ICD-10 code K86.0). In 2010 and 2011 the combination of these three ICD conditions accounted for 39,869 patients being admitted to hospitals (accounting for 52,316 admissions). The HES data will allow multiple admissions for the same patient to be identified through a pseudoanonymized identifier, and this will be considered in the analysis.

Examples of acute alcohol-related conditions include admissions for assault (ICD-10 code X85-Y09) and intentional self-harm (ICD-10 codes X60-X84 and Y10-Y34). These data will be extracted using the *Cause* field in HES. These admissions are not wholly attributable to alcohol (AAF=0.27 and AAF=0.34, respectively). A total of 119,360 patients were admitted to hospitals (accounting for 142,388 admissions) due to these causes in 2010 and 2011.

### Data on Confounding and Interaction

We plan to examine associations in several groups: young and older adults; men and women; areas with high, intermediate, and low levels of socioeconomic deprivation; and urban and rural areas. We will adjust for age (five-year bands) within the wider age groups. We will use the Index of Multiple Deprivation Income Domain as the indicator of socioeconomic deprivation at the small area level [34]. Population denominators will be obtained from the Office for National Statistics.

### Statistical Analyses

The relationship between alcohol outlet density and alcohol-related hospital admissions is complex, and is mediated through a range of factors including *supplier side* factors (eg, price and marketing) and individual level factors (eg, attitudes about alcohol, drinking behaviors, and demand for alcohol). These largely unobserved factors in ecological analyses may induce spatial and temporal correlation in outlet density and hospital admissions, and statistical models must account for these correlations if parameters are to be properly estimated. We propose to use Bayesian hierarchical modelling methodology, including a structural equation model framework in which we represent relationships between observed and unobserved *latent* variables, and in which we allow model errors to have spatial and temporal autocorrelation. Bayesian spatial models may be computationally intensive, and we have access to grid computing facilities for this purpose.

In the ecological correlation analyses, in which spatial associations will be examined, we will use LSOAs as the units of analysis, taking into account spatial autocorrelation (ie, the statistical nonindependence of neighboring geographic areas) and using an adjacency matrix in which LSOAs with common boundaries are classified as neighbors. Nonlinear associations will be investigated as increases in availability of alcohol outlets may have diminishing effects on alcohol-related harm as baseline availability increases [22]. This aspect is relevant, as our current MRC/ESRC work indicates that outlet density in the United Kingdom is generally much higher than that of other countries.

When examining changes over time, we plan to use the cross-sectional time series panel study methodology that has been used previously [7,17,18]. We aim to implement this methodology within a Bayesian framework, using MSOAs as the spatial units of analysis and years as time points. This methodology will allow for control of unmeasured spatial confounders and general time trends in hospital admission rates. The spatio-temporal methods will also allow for the examination of lag effects between exposure and outcome; lag time may be an important variable. For example, Stockwell et al observed that alcohol price changes exerted effects observable at zero lag for hospital admissions related to acute alcohol-related conditions, but these effects only became apparent from a two-year lag onwards for admissions related to chronic alcohol-related conditions [7].

Reverse causality hypotheses pose particular challenges in the interpretation of associations observed in analyses of outlet density and health outcomes. One hypothesis is that outlets cluster in *unhealthy* areas. To address this issue, we plan to investigate associations between outlet density and hospital admissions for conditions that would not be expected to be related to alcohol (eg, lung cancer and all emergency admissions unrelated to alcohol). Such conditions would reflect general influences on hospital admission, including the effects of area-level factors, provider units, and primary and community care, and should therefore control for these effects. A further reverse causality hypothesis is that low demand leads to a low density of alcohol outlets (as opposed to low outlet density resulting in low consumption levels, and therefore low alcohol-related hospital admissions). We will investigate whether this hypothesis can be ruled out by using cross-lagged models. These models are structural equation models in which, for example, the effects of two variables X and Y are measured at repeated time points (t1 and t2) and analyzed to investigate their effects on each other (ie, X1 on Y2 and Y1 on X2) [35].

## Results

The project is currently in progress. Results are expected in 2017.

## Discussion

If we do find associations, this study will provide the first contemporary England-specific evidence that changes in alcohol availability contribute to rates of alcohol-related harm. This finding would provide a useful evidence base to inform public health contributions and considerations for alcohol licensing decision-making. The results will allow local authorities to quantify the potential benefits of restricting outlet density on hospital admission rates. Alternatively, if we discover no evidence of association, this finding will be based on comprehensive national data. These data would support licensing authorities in not considering NHS costs due to hospital admissions as a priority in decision-making, in relation to outlet density. This finding would also add to the evidence base on appropriate approaches for reducing alcohol-related harm in England.

## Abbreviations

AAF: alcohol attributable fractions
HES: Hospital Episode Statistics
ICD-10: International Statistical Classification of Diseases and Related Health Problems-10
km: kilometer
LSOA: Lower Super-Output Area
MRC/ESRC: Medical Research Council/Economic and Social Research Council
MSOA: Middle Super-Output Area
NHS: National Health Service

## Appendix

Multimedia Appendix 1 Peer review of grant application (Reviewer 1).

Multimedia Appendix 2 Peer review of grant application (Reviewer 2).

## References

[1] Popova S, Giesbrecht N, Bekmuradov D, Patra J (2009). Hours and days of sale and density of alcohol outlets: impacts on alcohol consumption and damage: a systematic review. Alcohol Alcohol.

[2] Campbell CA, Hahn RA, Elder R, Brewer R, Chattopadhyay S, Fielding J, Naimi TS, Toomey T, Lawrence B, Middleton JC, Task Force on Community Preventive Services (2009). The effectiveness of limiting alcohol outlet density as a means of reducing excessive alcohol consumption and alcohol-related harms. Am J Prev Med.

[3] Bryden A, Roberts B, McKee M, Petticrew M (2012). A systematic review of the influence on alcohol use of community level availability and marketing of alcohol. Health Place.

[4] Holmes J, Guo Y, Maheswaran R, Nicholls J, Meier P, Brennan A (2014). The impact of spatial and temporal availability of alcohol on its consumption and related harms: a critical review in the context of UK licensing policies. Drug Alcohol Rev.

[5] Tatlow JR, Clapp JD, Hohman MM (2000). The relationship between the geographic density of alcohol outlets and alcohol-related hospital admissions in San Diego County. J Community Health.

[6] Livingston M (2011). Alcohol outlet density and harm: comparing the impacts on violence and chronic harms. Drug Alcohol Rev.

[7] Stockwell T, Zhao J, Martin G, Macdonald S, Vallance K, Treno A, Ponicki W, Tu A, Buxton J (2013). Minimum alcohol prices and outlet densities in British Columbia, Canada: estimated impacts on alcohol-attributable hospital admissions. Am J Public Health.

[8] Fone D, Morgan J, Fry R, Rodgers S, Orford S, Farewell D, Dunstan F, White J, Sivarajasingam V, Trefan L, Brennan I, Lee S, Shiode N, Weightman A, Webster C, Lyons R (2016). Change in alcohol outlet density and alcohol-related harm to population health (CHALICE): a comprehensive record-linked database study in Wales. Southampton (UK): NIHR Journals Library.

[9] Richardson EA, Hill SE, Mitchell R, Pearce J, Shortt NK (2015). Is local alcohol outlet density related to alcohol-related morbidity and mortality in Scottish cities?. Health Place.

[10] Department of Health and North West Public Health Observatory (2008). Hospital admissions for alcohol-related harm: understanding the dataset.

[11] Department of Health (2008). The cost of alcohol harm to the NHS in England. An update to the Cabinet Office (2003) study: Health Improvement Analytical Team.

[12] (2005). Licensing (Scotland) Act 2005 asp 16.

[13] HM Government (2012). The Government's Alcohol Strategy Cm 8336.

[14] SHAAP Alcohol Focus Scotland (2011). Re-thinking alcohol licensing.

[15] Colón I (1981). Alcohol availability and cirrhosis mortality rates by gender and race. Am J Public Health.

[16] Theall KP, Scribner R, Cohen D, Bluthenthal RN, Schonlau M, Lynch S, Farley TA (2009). The neighborhood alcohol environment and alcohol-related morbidity. Alcohol Alcohol.

[17] Stockwell T, Zhao J, Macdonald S, Vallance K, Gruenewald P, Ponicki W, Holder H, Treno A (2011). Impact on alcohol-related mortality of a rapid rise in the density of private liquor outlets in British Columbia: a local area multi-level analysis. Addiction.

[18] Zhao J, Stockwell T, Martin G, Macdonald S, Vallance K, Treno A, Ponicki WR, Tu A, Buxton J (2013). The relationship between minimum alcohol prices, outlet densities and alcohol-attributable deaths in British Columbia, 2002-09. Addiction.

[19] Chiang C (2010). Review of the Relationship between Outlet Density and Crime in NHS Greater Glasgow and Clyde.

[20] Langley J, Bellamy V (2011). Pub, club and off-licence density in the East Midlands.

[21] Gruenewald P (2008). Why do alcohol outlets matter anyway? A look into the future. Addiction.

[22] Livingston M, Chikritzhs T, Room R (2007). Changing the density of alcohol outlets to reduce alcohol-related problems. Drug Alcohol Rev.

[23] Hough M, Hunter G (2003). The 2003 Licensing Act's impact on crime and disorder: an evaluation. Criminology Crim Justice.

[24] Gruenewald P (2007). The spatial ecology of alcohol problems: niche theory and assortative drinking. Addiction.

[25] Erskine S, Maheswaran R, Pearson T, Gleeson D (2010). Socioeconomic deprivation, urban-rural location and alcohol-related mortality in England and Wales. BMC Public Health.

[26] Office for National Statistics (2009). General Lifestyle Survey Overview: A report on the 2009 General Lifestyle Survey.

[27] (1999). National Travel Survey 1997/9 Update.

[28] Competition Commission (2008). The supply of groceries in the UK market investigation.

[29] Brindley P, Wise SM, Maheswaran R, Haining RP (2005). The effect of alternative representations of population location on the areal interpolation of air pollution exposure. Comput Environ Urban Syst.

[30] (2016). CGA Strategy.

[31] Health and Social Care Information Centre (2016). NHS Digital.

[32] Public Health England (2014). User Guide: Local Alcohol Profiles for England.

[33] Jones L, Bellis M (2013). Updating England Specific Alcohol-Attributable Fractions.

[34] Department for Communities and Local Government (2012). English indices of deprivation.

[35] Finkel S (1995). Causal analysis with panel data.

