# Supplementary material for: Investigation of the Association Between Alcohol Outlet Density and Alcohol-Related Hospital Admission Rates in England: Study Protocol
Source: JMIR Res Protoc. 2016 Dec 16;5(4):e243. doi: 10.2196/resprot.6300 (PMC5203678; doi:10.2196/resprot.6300)
Supplement: Multimedia Appendix 1 [file resprot_v5i4e243_app1.pdf]

Alcohol Research UK
Research and Development Grants Programme 2014

Thank you for agreeing to review this application. Please complete the form below, referring to our research priorities attached, and provide your comments on the proposal.

APP 56 2014: ScHARR, UNIVERSITY OF SHEFFIELD
Peer Review 1
'Investigation of the association between alcohol outlet density and alcohol related hospital admission rates in England'

CRITERIA:	Excellent	Good	Satisfactory	Unsatisfactory
1. How clearly does the proposal align with our current research priorities?	✓			
2. How achievable/feasible is the project likely to be/how adequate is the project planning?		✓		
3. Is the proposed methodology sound?		✓		
4. How impactful is the project likely to be?		✓		
5. Does the project offer good value for money?		✓		

COMMENTS:

Please comment on any technical issues regarding feasibility, project planning or methods:

The research team have the necessary expertise for undertaking this project and have a strong history of producing high quality work in the field. The project methods and planning have been well considered and appear thorough and comprehensive. I just have a few issues that may affect the project outlined below:

- Licensing data completeness may affect the findings from the study. The application states that the 2010 CGA dataset contains 184964 alcohol outlets, estimated to be 98% of all those in England. Official statistics for 2010 record 202000 premises licenses with data provided from 96% of local authorities. It may be that certain types of outlets are missing from the CGA dataset and it would be useful if some verification of density could be undertaken, even at a small area level.
- The research team likely have experience of accessing hospital admissions data and may have already obtained agreement access to this, but from my own recent experience there are currently strict controls on what data are being made available to external bodies including restrictions on the provision of small area data and linkage of data items. This may represent a risk to the project – although I don't think it should affect the funding decision.
- I am presuming hospital admissions data will be based on patients' residential LSOA? For analyses of on-licensed premises in particular, this may create some issues as individuals travel to town and city centres for nights out and population density in areas where pubs

and nightclubs are concentrated can be low. The effects of this would need to be taken into consideration when interpreting findings

- I think the issues around supermarkets may be worth examining in more detail if this is possible. It is likely true that they will not assert a strong density type effect given their reach across the population. However, they can provide much more alcohol in terms of quantity than say a small convenience store, can offer alcohol more cheaply and can represent a greater push towards alcohol purchase for consumers (e.g. multi-buy promotions). They can also push smaller stores out of business – so the opening of a single Tesco Metro or similar might lead to the closure of one or more existing off-licences or convenience stores in the immediate neighbourhood that will therefore lead to reduced alcohol outlet density yet greater local alcohol availability. I believe CGA include alcohol sales data and it may be possible for the team to control for this - however findings around differential impacts on alcohol-related harms will need to be reported with caution.
- Updated attributable fractions for England have just been published, although I'm sure the research team will be aware of this already.

Any other comments:

A generally sound proposal that will make a useful contribution to UK understanding on alcohol licensing issues. To my knowledge, there are no previous national studies of the relationship between alcohol outlet density and alcohol-related health harms in England yet local agencies increasingly need such information in order to inform the implementation of cumulative impact policies and other licensing matters.
