# Supplementary material for: Investigation of the Association Between Alcohol Outlet Density and Alcohol-Related Hospital Admission Rates in England: Study Protocol
Source: JMIR Res Protoc. 2016 Dec 16;5(4):e243. doi: 10.2196/resprot.6300 (PMC5203678; doi:10.2196/resprot.6300)
Supplement: Multimedia Appendix 2 [file resprot_v5i4e243_app2.pdf]

Alcohol Research UK
Research and Development Grants Programme 2014

Thank you for agreeing to review this application. Please complete the form below, referring to our research priorities attached, and provide your comments on the proposal.

APP 56 2014: ScHARR, UNIVERSITY OF SHEFFIELD
Peer Review 2
'Investigation of the association between alcohol outlet density and alcohol related hospital admission rates in England'

CRITERIA:	Excellent	Good	Satisfactory	Unsatisfactory
1. How clearly does the proposal align with our current research priorities?	x			
2. How achievable/feasible is the project likely to be/how adequate is the project planning?		x		
3. Is the proposed methodology sound?		x		
4. How impactful is the project likely to be?			x	
5. Does the project offer good value for money?		x		

COMMENTS:

Please comment on any technical issues regarding feasibility, project planning or methods:
The authors have not indicted whether access to the HES data is guaranteed and what alternative options if the data is delayed or not available.

The dissemination strategy is not very detailed nor comprehensive. The target audiences of local authorities in terms of planning and licensing controls and alcohol related NGOs in terms of harm reduction are not identified.

The project does not discuss the limitation of the assumption that consumption is generally close to admission. Where this may be expected to be the case in terms of acute admissions those for chronic diseases will be expected at the home address. Using more coarse geographies may offer some solution in urban areas, but drinking in for example London and residence in the south east would still be missed. If no association is found this may not be due to here being no relationship. The project being ecological also is at risk of the modifiable area unit problem and associations at one geography may not be causal.

Any other comments:

The £2000 for open access publication should not be awarded as universities have directly awarded funds for this purpose.

£2000 for conference and other travel is high for a part time 18 month project. The conferences and the number of attendees at each need to be identified to justify the funding.
